# Multi locus sequence typing of clinical *Burkholderia pseudomallei* isolates from Malaysia

**DOI:** 10.1371/journal.pntd.0008979

**Published:** 2020-12-28

**Authors:** Revathy Arushothy, Fairuz Amran, Nazirah Samsuddin, Norazah Ahmad, Sheila Nathan

**Affiliations:** 1 Institute for Medical Research, National Institutes of Health, Shah Alam, Selangor, Malaysia; 2 Department of Biological Sciences and Biotechnology, Faculty of Science and Technology, Universiti Kebangsaan Malaysia, Bangi, Selangor, Malaysia; Colorado State University, UNITED STATES

## Abstract

**Background:**

Melioidosis is a neglected tropical disease with rising global public health and clinical importance. Melioidosis is endemic in Southeast Asia and Northern Australia and is of increasing concern in Malaysia. Despite a number of reported studies from Malaysia, these reports are limited to certain parts of the country and do not provide a cohesive link between epidemiology of melioidosis cases and the nation-wide distribution of the causative agent *Burkholderia pseudomallei*.

**Methodology/principle findings:**

Here we report on the distribution of *B*. *pseudomallei* sequence types (STs) in Malaysia and how the STs are related to STs globally. We obtained 84 culture-confirmed *B*. *pseudomallei* from confirmed septicaemic melioidosis patients from all over Malaysia. Prior to performing Multi Locus Sequence Typing, the isolates were subjected to antimicrobial susceptibility testing and detection of the YLF/BTFC genes and BimA allele. Up to 90.5% of the isolates were sensitive to all antimicrobials tested while resistance was observed for antimicrobials typically administered during the eradication stage of treatment. YLF gene cluster and *bimA*_Bp_ allele variant were detected in all the isolates. The epidemiological distribution patterns of the Malaysian *B*. *pseudomallei* isolates were analysed *in silico* using phylogenetic tools and compared to Southeast Asian and world-wide isolates. Genotyping of the 84 Malaysian *B*. *pseudomallei* isolates revealed 29 different STs of which 6 (7.1%) were novel. ST50 was identified as the group founder followed by subgroup founders ST376, ST211 and ST84. A low-level diversity is noted for the *B*. *pseudomallei* isolates described in this study while phylogenetic analysis associated the Malaysian STs to Southeast Asian isolates especially isolates from Thailand. Further analysis also showed a strong association that implicates agriculture and domestication activities as high-risk routes of infection.

**Conclusions/significance:**

In conclusion, MLST analysis of *B*. *pseudomallei* clinical isolates from all states in Malaysia revealed low diversity and a close association to Southeast Asian isolates.

## Author summary

*Burkholderia pseudomallei*, a Gram-negative saprophytic bacterium, is the causative agent of melioidosis. The burden of human melioidosis globally is predicted at 165,000 cases and 89,000 deaths annually and in Malaysia, it is estimated that more than 2000 patients die per year which is much higher than nation-wide deaths resulting from dengue or tuberculosis. Clinical presentation and antibiotic resistance vary by geographical regions making it difficult for public health officials to outline definitive diagnostic, treatment and outbreak management options for the country. This study provides information on the genetic diversity among Malaysian *B*. *pseudomallei* clinical isolates. The epidemiological analysis shows strong correlation of the Malaysian isolates with strains from Southeast Asia especially Thailand, reflecting the regional endemicity.

## Introduction

Melioidosis is recognized as an emerging global problem and is an endemic disease of public health and clinical importance in tropical and subtropical regions of the world. Melioidosis results from an infection by the Gram negative saprophytic bacterium *Burkholderia pseudomallei* [1,2]. Infection occurs through skin and by inhalation when susceptible individuals are exposed to contaminated water and soil [2–5]. Due to its high virulence, *B*. *pseudomallei* is classified as a Tier 1 select agent by the U.S. Centers for Disease Control and Prevention (US CDC) [6] (http://www.selectagents.gov/).

The burden of human melioidosis is estimated at almost 165,000 cases annually with a mortality rate of over 50%, a burden similar to that of measles [7]. Multiple cases of melioidosis have been reported in India and several countries within South-East Asia, the Middle East, Africa and South America [7,8]. Melioidosis has been reported to account for about 20% of all community-acquired septicemias in north-eastern Thailand and 2000 to 3000 new cases are diagnosed every year [7].

In Malaysia, based on known incidence and mortality due to melioidosis, it is estimated that more than 2000 patients die per year, which is much higher than death resulting from dengue or tuberculosis [9]. Despite many reports of melioidosis cases throughout Peninsular Malaysia, based on reports from Pahang, Kedah, Kelantan and Johor, as well as from Sarawak and Sabah, which make up East Malaysia, the actual disease burden is unknown since melioidosis is not notifiable under the Prevention and Control of Communicable Diseases Act of 1988 (Act 342). Recently, states with high incidence such as Pahang and Sabah have come up with their own registry and guidelines [10–12].

This uncertainty in the true number of cases and fatalities attributed to melioidosis in Malaysia is related to the lack of awareness of the disease among primary healthcare professionals and the general public. As such, a country-wide survey of *B*. *pseudomallei* isolated from melioidosis patients using molecular methods such as sequence typing will be useful to assess the population structure of the organism locally as well as in relation to the worldwide *B*. *pseudomallei* population [13,14]. On a broader scope, molecular epidemiology of *B*. *pseudomallei* will also provide insights into the biogeography and factors that influence the distribution of the organism within a particular ecology where such knowledge can assist in public health management during outbreaks [15,16]. A recent report from Malaysia [17] has revealed genetic diversity among the clinical *B*. *pseudomallei* isolates from one state in the north-east of Malaysia.

To fill in the gap on epidemiology of melioidosis in the country and to be able to assess the relatedness of the Malaysian isolates with those described in neighboring endemic hotspots, we undertook a study on *B*. *pseudomallei* clinical isolates contributing to septicaemic melioidosis in Malaysia to provide information on the circulating sequence types (ST) especially in highly endemic areas, and to determine how *B*. *pseudomallei* from Malaysia is related to strains found elsewhere.

## Methods

### Ethics statement

The Malaysian Research Ethics Committee and National Medical Research Registry approved this study under the protocol number NMRR- 18-805-41423.

### Bacterial isolates

**Fig 1:**
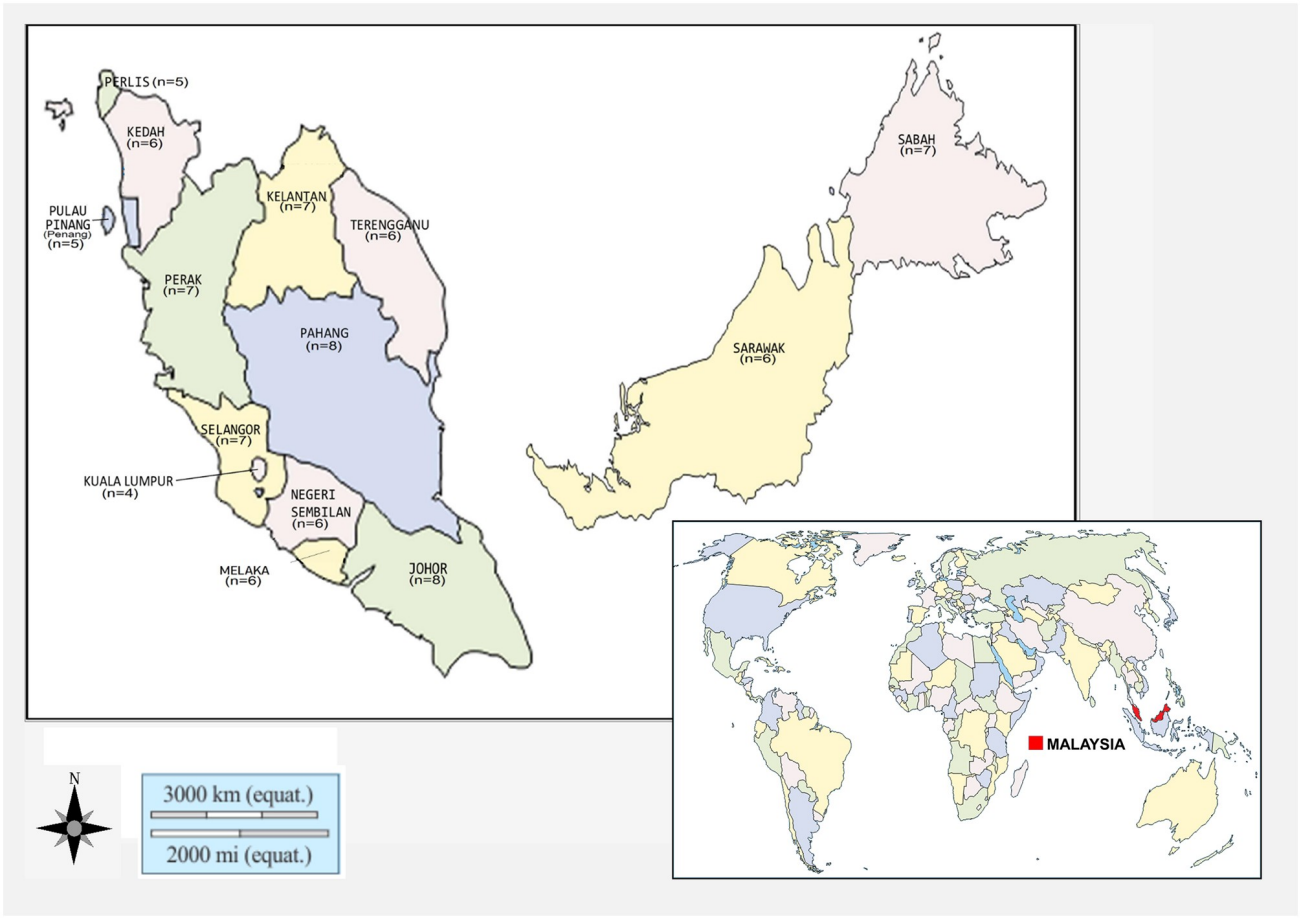
The map of the world indicates the location of Malaysia while the map of Malaysia identifies the states in Malaysia from which *B*. *pseudomallei* isolates were obtained and the number of isolates from each state. The figure was recreated using the open source software https://d-maps.com/carte.php?num_car=126802&lang=en and https://d-maps.com/pays.php?num_pay=97&lang=en.

The National Surveillance for Antibiotic Resistance Malaysia documented approximately 1500 *B*. *pseudomallei* isolates per year between 2017 and 2019. Interestingly, 60% of these isolates were from septicaemic infection. Despite the availability of this information, the true incidence of melioidosis and eventual patient outcome is not known as notification of the disease is not mandatory in Malaysia. In this study, we obtained eighty-four (n = 84) *B*. *pseudomallei* clinical isolates from patients confirmed to have melioidosis or septicaemia, from tertiary government hospitals throughout Malaysia. The number of isolates obtained from individual states in Malaysia is Perlis 5, Kedah 6, Pulau Pinang (Penang) 5, Perak 7, Selangor 7, Federal Territory of Kuala Lumpur 4, Melaka 6, Negeri Sembilan 6, Johor 8, Terengganu 6, Kelantan 7, Pahang 8, Sabah 7 and Sarawak 6 (Fig 1). These isolates were submitted to the Institute for Medical Research, Kuala Lumpur between July to October 2019. All isolates were confirmed using the Vitek 2 GN card (BioMérieux, France) and with 16S rRNA amplification.

The isolates were subjected to antimicrobial susceptibility screens. Minimum Inhibitory Concentrations (MICs) for amoxicillin-clavulanic acid, ceftazidime, imipenem, doxycycline, tetracycline and trimethoprim-sulfamethoxazole were determined using E-tests (bioMérieux, France) according to manufacturer’s instructions. Amoxicillin-clavulanic acid, ceftazidime, imipenem, doxycycline, tetracycline and trimethoprim-sulfamethoxazole were selected for MIC determination since these antibiotics are used in routine melioidosis treatment. Breakpoints were interpreted using the Clinical & Laboratory Standards Institute [CLSI, 2017] guidelines. *Escherichia coli* ATCC 25922 and *Pseudomonas aeruginosa* ATCC 27853 were used as controls.

### Determination of epidemiological markers

The isolates were subjected to epidemiological marker screens such as lipopolysaccharide (LPS) profiling, determination of the *Burkholderia thailandensis* like flagellum (BTFC) and *Yersinia* like fimbriae (YLF) genes and detection of the BimA allele variant. Total genomic DNA was extracted using the DNAeasy Blood and Tissue DNA kit (Qiagen Gmbh, Hilden), according to the manufacturer’s instructions for Gram-positive bacteria.

Multiplex PCR assay was optimized to detect LPS A, LPS B and LPS B2 [18] and BTFC and YLF genes [19]. All primers used in the multiplex PCR assays are listed in Table 1.

**Table 1:** List of primers used for the determination of epidemiological markers.

Gene	Primer Pairs	Expected Amplicon Size (bp)
**LPS A**	F-TCAAACCTATCCGCGTGTCGAAGT R-TCGTCGTCAAGAAATCCCAGCCAT	195
**LPS B**	F-AATCTTTTTCTGATTCCGTCC R -ACCAGAAGACAAGGAGAAAGGCCA	93
**LPS B2**	F- AACCGGGTAGTTCGCGATTAC R-ATACGCCGGTGTAGAACAGTA	364
**BTFC**	F-TGTTTCGCAGCGAGGATGTC R- CCCACCGTCAAGCCGATT	115
**YLF**	F- GTGCCTGCAACGCTAATCG R-CGCACTGATAGCCGGAATAGAG	350
***bimA***_**Bp**_	F-GGAAGCTTTGGCGTGCATAT R-CCCATGCCTTCCTCGACTAAT	60
***bimA***_**Bm**_	F- AGCGCTTCGCGCATCTAC R- CGCGTTAAACGCCGTACTTTC	104

For LPS, the Multiplex PCR was performed using 2x MyTaq Red mix (Bioline Ltd UK) on the Master cycler gradient (Eppendorf, Hamburg, Germany) with an initial denaturation at 95°C for 10 min, followed by 30 cycles of 95°C for 30 sec, 58°C for 30 sec, 72°C for 30 sec and final extension step of 72°C for 7 min. For YLF and BTFC gene screens, the protocol involved 2x MyTaq Red mix (Bioline Ltd UK) with an initial denaturation at 95°C for 10 min, followed by 35 cycles of 95°C for 30 sec, 60°C for 30 sec, 72°C for 30 sec and final extension step of 72°C for 7 min.

The *bimA* variant for *B*. *pseudomallei* BimA allele (*bimA*_Bm_) and *B*. *mallei* BimA allele (*bimA*_Bm_) were identified using conventional PCR [20]. The PCR was performed using the primers listed in Table 1 and 2x MyTaq Red mix Bioline Ltd UK) in a Master cycler gradient (Eppendorf, Hamburg, Germany) with an initial denaturation at 95°C for 10 min, followed by 30 cycles of 95°C for 30 sec, 56°C for 30 sec, 72°C for 30 sec and final extension step of 72°C for 7 min.

All amplicons were subjected to agarose gel electrophoresis (100 V for 15 min) with a 100bp ladder and visualized using the Quantity One v4.62 gel documentation system (Bio-Rad Laboratories, Inc., Hercules, CA, USA).

### Multi locus sequence typing (MLST)

MLST was performed by amplification of seven *B*. *pseudomallei* housekeeping genes (*ace*, *gltB*, *gmhD*, *lepA*, *lipA*, *nark*, *ndh*) as described previously [21]. The primers used for the Polymerase Chain Reaction (PCR) were obtained from the *Burkholderia pseudomallei* database (http://pubmlst.org/bpseudomallei/). Amplification of the seven housekeeping genes was performed and the raw reads generated from the forward and reverse sequences of the seven housekeeping genes were visually reviewed and edited using the Chromas Lite 2.1 software (Technelysium, South Brisbane, Australia) and aligned with ClustalW within the MEGA7 software. The verified sequences of each respective housekeeping gene were analysed within the PubMLST *B*. *pseudomallei* isolates database to assign allelic numbers and defined sequence types (STs). Novel STs were assigned new allelic profile numbers and defined new STs. All sequence types are available in the PubMLST database.

### Global optimal eBURST (goeBURST) analysis

Genetic relatedness of the isolates was analysed using the goeBURST algorithm of the PHYLOVIZ open source software to establish epidemiological association among the *B*. *pseudomallei* isolates from different regions in Malaysia. goeBURST allows for an unrooted tree-based representation of the relationship between isolates and was used to determine the relationship between the Malaysian STs with the global collection of STs [22]. The clonal complexes were determined using the goeBURST output tab that provides information on the STs that make up each complex and the edges (links between STs) to derive the complexes (*https*:*//phyloviz*.*readthedocs*.*io/en/latest/data_analysis*.*html*).

A phylogenetic tree was constructed using Hierarchical Clustering with Unweighted Pair Group Method with Arithmetic average (UPGMA) method within PHYLOVIZ. The Malaysian isolates STs including newly identified STs were analysed with 30 selected STs from Thailand, Vietnam, India, Australia, China and Vietnam.

## Results

### Identification of *Burkholderia pseudomallei* isolates from all states in Malaysia

Eighty-four *B*. *pseudomallei* isolates were collected from hospitals all around Malaysia and sent to the Bacteriology Unit, Institute for Medical Research. The isolates were re-identified using Gram stain, oxidase test and Vitek 2 (GN card) (Biomerieux, France) to confirm the original identification (S1 Table). All the isolates were obtained from patients with confirmed septicemic melioidosis. The patients were between 7 to 92 years old with the majority (45.5%) between the ages of 35 to 64. *B*. *pseudomallei* was commonly isolated from males at 77.0% (65 isolates) in comparison to females, 23.0% (19 isolates). More than half of the isolates (57%, 47 isolates) were obtained from Malay individuals in comparison to Chinese, Indian and other ethnic groups in Malaysia. The occupations of the patients in the study are mainly related to agriculture or as driver/lorry driver, however this only encompasses 20% of the isolates as not all patients’ occupations could be obtained.

### Distribution of *Burkholderia pseudomallei* STs in Malaysia

**Fig 2:**
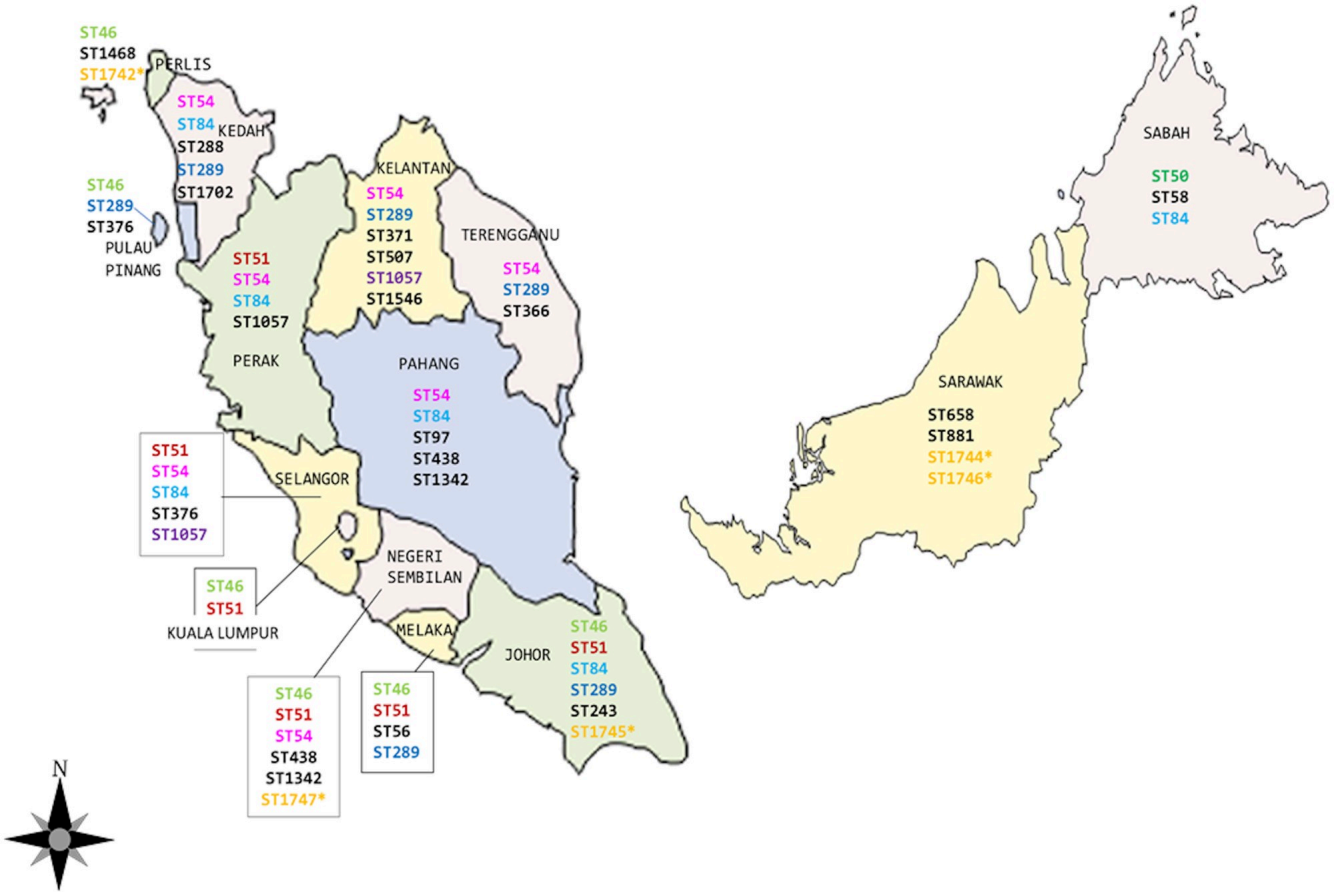
Distribution of *B*. *pseudomallei* Sequence Types (STs) according to states in Malaysia and its association with the globally distributed STs retrieved from the PubMLST database. Light green (ST46-Australia, Bangladesh, Southeast Asia,); Dark green (ST50- China, Thailand); Red (ST51-China, Thailand, Singapore); Pink (ST54-China, Thailand, Singapore); Light Blue (ST84- Australia, Singapore, Thailand); Dark Blue (ST289-Singapore, Thailand); Purple (ST1057-Singapore, Thailand); Yellow* (Novel STs from this study). This figure was recreated using the open source software https://d-maps.com/carte.php?num_car=26371&lang=en.

Among the 84 isolates in this study, a total of 29 sequence types (STs) were identified (Table 2). The frequency of STs among the isolates ranged from 1 to 12 with ST84 (n = 12), ST54 (n = 12), ST46 (n = 10), ST51 (n = 8), ST289 (n = 6), ST1057 and ST1342 (n = 4) being the most dominant STs. Six STs (ST1742, ST1743, ST1744, ST1745, ST1746 and ST1747) have not been previously reported. Information on the isolates is presented in Table 2 and distribution of the STs in the different states of Malaysia is plotted in Fig 2.

**Table 2:** Distribution of sequence types (ST) across Malaysia.

Sequence Type	Number of isolates	Location (States)
ST84	12	Kedah, Perak, Selangor, Johor, Pahang
ST54	12	Kedah, Perak, Selangor, Kelantan, Negeri Sembilan, Terengganu, Pahang
ST46	10	Perlis, Pulau Pinang, Kuala Lumpur, Melaka, Johor
ST51	8	Perak, Selangor, Kuala Lumpur, Negeri Sembilan, Melaka, Johor
ST289	6	Kedah, Pulau Pinang, Kelantan, Melaka, Johor, Terengganu
ST1057	4	Perak, Selangor, Kelantan
ST1342	4	Negeri Sembilan, Pahang
ST50	3	Sabah
ST658	3	Sarawak
Others	22	Distributed in all states in Malaysia

### Genetic relatedness among STs from Malaysia and its global association

**Fig 3:**
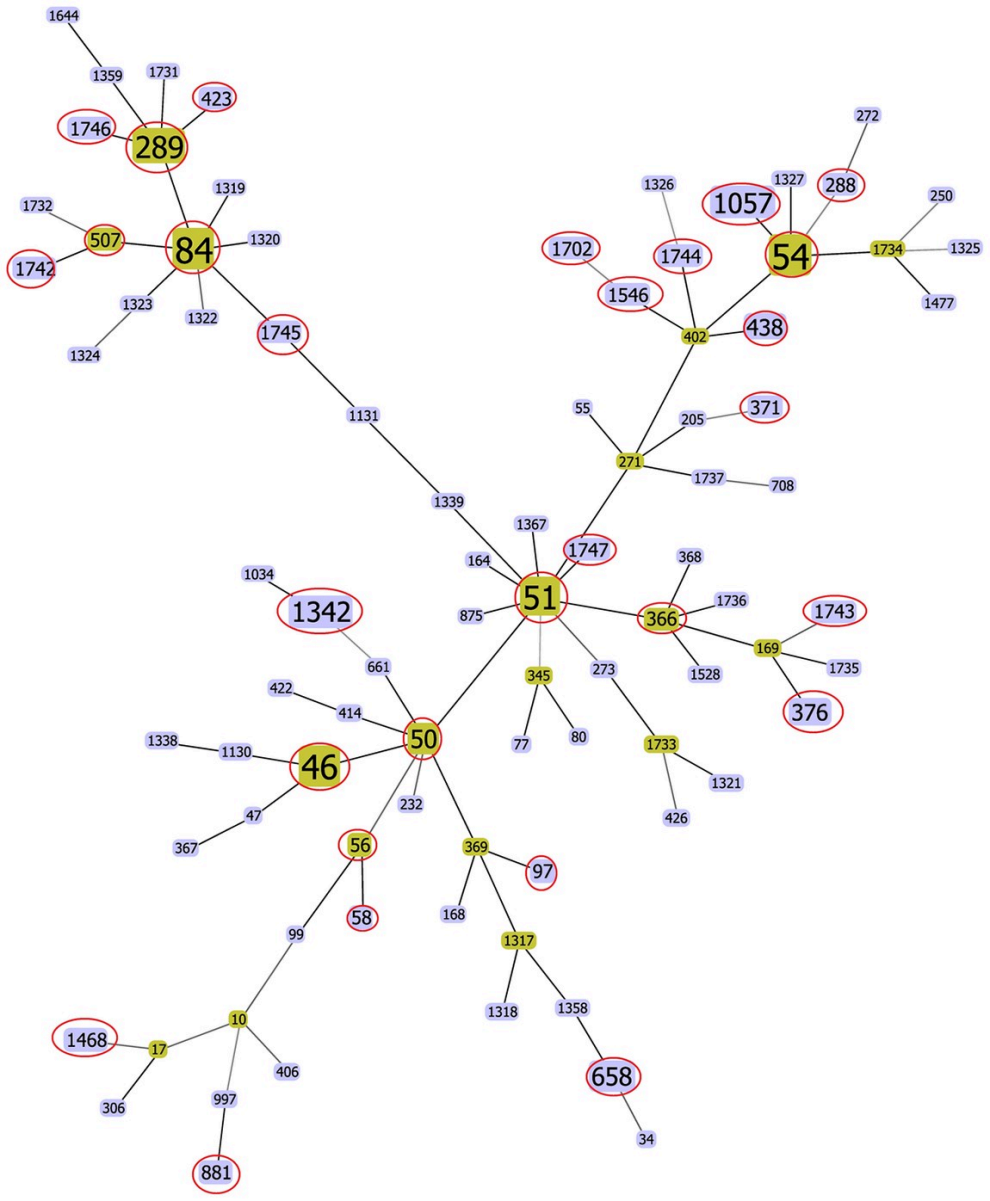
FullMST of 81 *B*. *pseudomallei* STs from Malaysia based on goeBURST analysis. STs of isolates from this study are circled in red.

Information on the 84 isolates from this study was deposited into the pubMLST database bringing the total of *B*. *pseudomallei* isolates from Malaysia to 536. The number of STs reported from Malaysia is 81 of which 29 STs are from this study (S2 Table). All 81 STs from Malaysia were subjected to goeBURST analysis using the PHYLOViz software [22] and the full minimum spanning tree (MST) (Fig 3) showed that the STs were clustered into five major complexes. The major complexes comprised of 56 STs and ST51 as the predicted group founder, followed by subgroup founders ST46, ST50, ST54, ST84 and ST289. Twenty-three STs from this study have been reported by other studies conducted in Australia, Bangladesh and other Southeast Asian countries.

**Fig 4:**
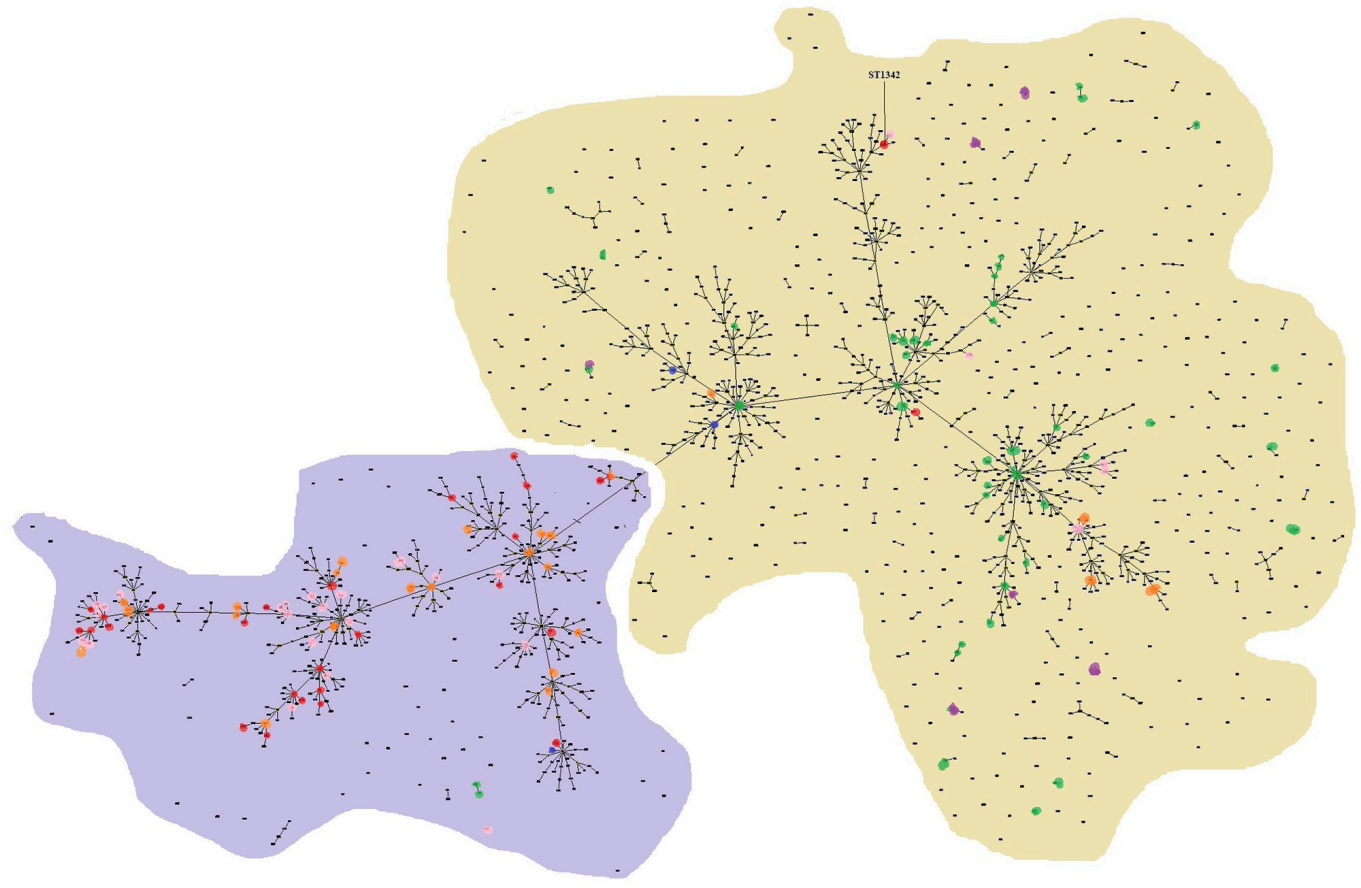
Genetic relationship among the global collection of STs available in the PubMLST database using goeBURST analysis. Each dot represents one ST. Southeast Asia is shaded in purple and other endemic countries from Oceania (Australia and Papua New Guinea) are shaded in yellow. The colour codes for STs are: red dots—isolates from this study, pink dots—other Malaysian isolates, orange dots—Thailand, green dots—Australia and purple dots—Papua New Guinea.

A population structure analysis was also performed using the available 1643 STs present in the PubMLST database (Fig 4). These include 29 STs from this study (pink dots) and other STs (red dots) identified from Malaysia. This analysis resulted in 633 clonal complexes (CC), in which a large number of STs common to Malaysia lie within the largest clonal complex comprising of 1078 STs.

**Fig 5:**
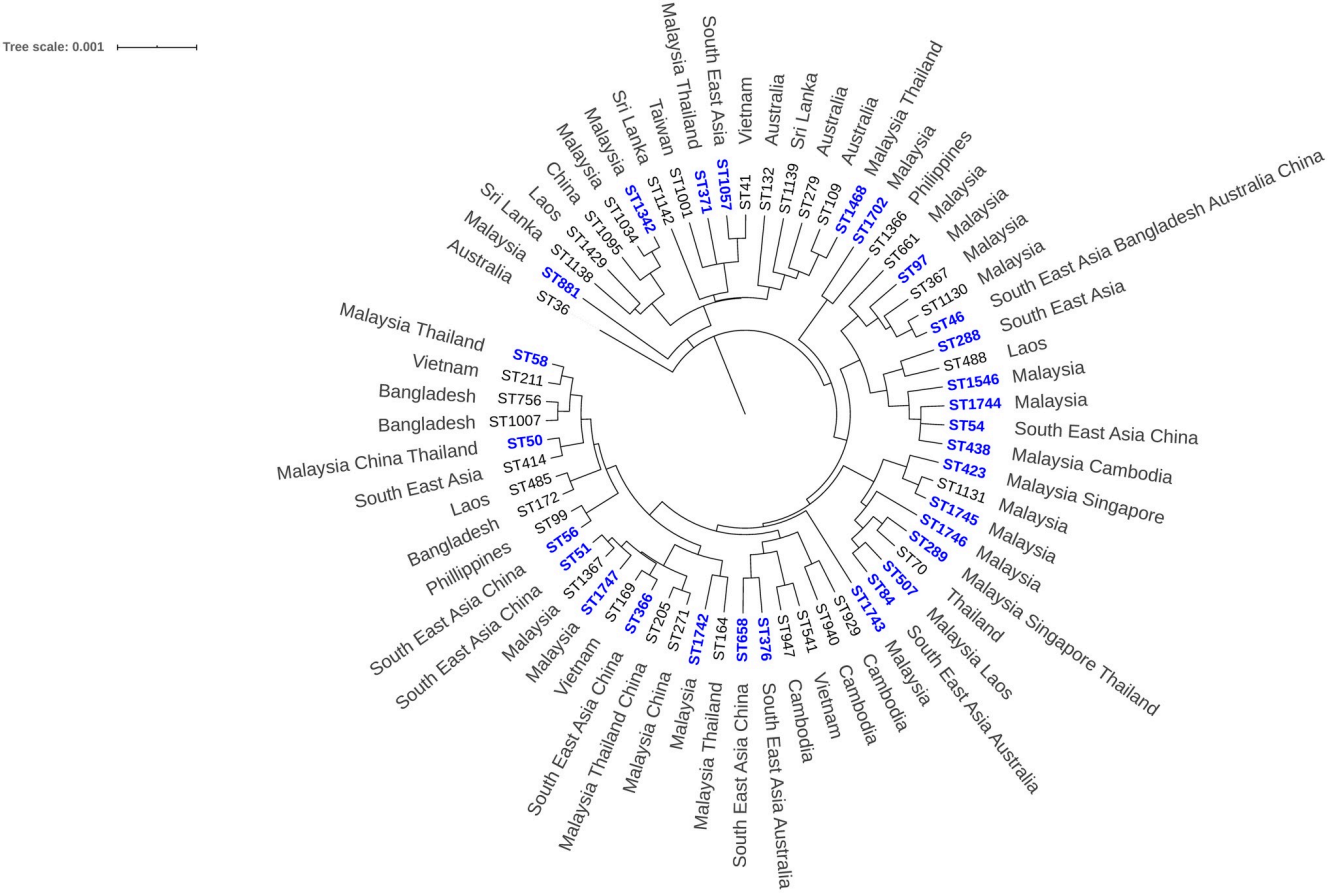
The evolutionary history inferred using UPGMA. The analysis involved 29 STs from this study with 36 other STs previously reported from other countries. The STs in blue represent STs of isolates from this study. Southeast Asian countries include Malaysia, Singapore, Indonesia, Thailand, Vietnam, Myanmar and the Philippines.

The topology and grouping of STs from Malaysia and other global collections are shown in Fig 5. Some of the STs included in this analysis are previously isolated *B*. *pseudomallei* from environmental and animal sources. These STs include ST1034, ST661, ST367, ST1130, ST1131, ST164, ST271 and ST205. The full MST construction comprising of the Malaysian isolates and other common isolates from melioidosis endemic countries shows that the STs from this study are closely clustered to the Asian STs. Furthermore, all isolates are YLF positive, a gene cluster found predominantly among isolates of Southeast Asian origin. All the isolates were also *B*. *pseudomallei* BimA allele variant (*bimA*_Bp_), a further linkage to Southeast Asia [23]. All *B*. *pseudomallei* isolates identified in this study are LPS type A with the exception of ST1342 isolated from Negeri Sembilan and Pahang which is LPS type B (S2 Table). The BTFC gene was not detected in any of the isolates.

### Correlation between antimicrobial screening and STs

The isolates were subjected to E-tests to determine the Minimum Inhibitory Concentrations (MICs) for amoxicillin-clavulanate, ceftazidime, imipenem, doxycycline, tetracycline and trimethoprim-sulfamethoxazole (S2 Table). Seventy-six (90.5%) isolates were sensitive to all drugs tested. Only three (3.6%) *B*. *pseudomallei* isolates, BP39 (ST51), BP64 (ST289) and BP210 (ST46), were resistant to three antimicrobial agents which were amoxicillin-clavulanate, tetracycline and trimethoprim-sulfamethoxazole. Four (4.8%) isolates resistant to only trimethoprim-sulfamethoxazole are BP19 (ST658), BP63 (ST289), BP165 (ST376) and BP193 (ST658). BP14 (ST289) was the only isolate resistant to tetracycline. The MIC_90_ of the isolates are within the sensitive range (Table 3).

**Table 3:** MIC_90_ of *B*. *pseudomallei* isolates from this study.

Antibiotic	MIC_90_ range	Antibiotic	MIC_90_ range
Amoxicillin Clavulanate	1.00–4.00 μg/ml	Tetracycline	1.00–8.00 μg/ml
Ceftazidine	0.75–3.00 μg/ml	Trimethoprim-sulfamethoxazole	0.016–2.00 μg/ml
Imipenem	0.19–0.75 μg/ml	Doxycycline	0.38–1.50 μg/ml

## Discussion

*Burkholderia pseudomallei* is a Gram-negative saprophytic bacterium classified as a Tier 1 Biological Select Agent. *B*. *pseudomallei* is the causative agent of melioidosis, a disease of increasing burden in Malaysia [9]. Nevertheless, published reports of disease incidence in Malaysia are sporadic and the available data do not allow us to associate any meaningful relationship between the epidemiology of *B*. *pseudomallei* circulating within the highly endemic communities in Malaysia and the actual disease burden. This study is the first to collect *B*. *pseudomallei* isolates causing septicaemic infection from all the states in Malaysia, which were then analyzed to determine the epidemiological distribution.

The *B*. *pseudomallei* isolates were obtained from blood cultures of patients initially diagnosed with melioidosis, septicaemia or pneumonia and later confirmed as septicaemic melioidosis. We chose to limit the study to only blood culture isolates as these contributed to the highest number of *B*. *pseudomallei* isolates recorded in The National Surveillance for Antibiotic Resistance Malaysia [24]. The *B*. *pseudomallei* isolates in this study were primarily from septicaemic melioidosis patients aged between 35 to 64 years old (mean age: 50 years old). This is also the age group where co-morbidities such as diabetes are detected in melioidosis patients [9,25]. The *B*. *pseudomallei* from this study were more frequently isolated from males of Malay ethnicity with agriculture or farming as the dominant occupation. Hassan et al. [26] also reported similar demographics for *B*. *pseudomallei* clinical cases in the state of Kedah, Malaysia. In Malaysia, there is a higher tendency for males to be involved in soil and agriculture-based occupations and activities that may have facilitated exposure to the bacterium.

MLST is an unambiguous and powerful procedure to study bacterial populations and global epidemiology [21]. It has been extensively utilized to characterize *B*. *pseudomallei* and analysis of MLST data has succeeded in demarking genetic relatedness of *B*. *pseudomallei* from distinct geographical locations. The 84 clinical isolates of *B*. *pseudomallei* collected from all the states in Malaysia were characterized by MLST to understand the genotypic diversity and relatedness to other *B*. *pseudomallei* isolates from Asia or Australia. Twenty-nine STs were identified and overall diversity of the isolates was 0.35 STs/isolate. This shows that the diversity of the *B*. *pseudomallei* isolates in Malaysia is considerably lower when compared with a diversity ratio of 0.65 STs/isolate reported in Australia [27]. Previous ST analysis for *B*. *pseudomallei* [17] in Malaysia also showed similar diversity which was 0.38 STs/isolate. Other studies from Thailand [28] also showed a very low diversity with only 7 sequence types identified from 630 *B*. *pseudomallei* isolates.

The common sequence types (STs) were ST84 (14.3%), ST54 (14.3%). ST46 (11.9%), ST51 (9.5%) and ST289 (7.1%). The distribution of the STs throughout Malaysia is between 3 to 5 STs per state with ST84, ST54 and ST46 being most common in all states in Peninsular Malaysia and Sabah. However, in Sarawak, ST658, ST881, ST1744 and ST1746 were more common (Fig 2). A previous report also described that *B*. *pseudomallei* from Sarawak are phenotypically different in terms of sensitivity to aminoglycosides and gentamicin [29]. The PubMLST *B*. *pseudomallei* database search showed that the common STs in Malaysia such as ST46, ST50, ST51, ST54, ST84, ST289 and ST1057 are also common in countries such as Australia and Bangladesh and most common in Southeast Asian countries.

Four clonal complexes were identified from the goeBURST analysis with ST50 as group founder and ST54, ST84 and ST211 as subgroup founders. Three novel STs from this study (ST1742, ST1745, and ST1746) descended from ST84. Other novel STs, ST1743, ST1744 and ST1747 are spread over other complexes. Analysis of the STs from this study and previously reported STs in PubMLST pointed to a strong association with *B*. *pseudomallei* isolates from Thailand. This suggests that STs of clinical *B*. *pseudomallei* strains from the four complexes in Malaysia are dominant within the region and the expansion of the local STs yielded new isolates with novel STs.

Clustering of the local *B*. *pseudomallei* clinical isolates with STs reported from local animal and environmental isolates recorded in the PubMLST database and with other Asian countries and Australia also suggests that there is a genetic relatedness of Malaysian *B*. *pseudomallei* isolates with isolates from countries in Southeast Asia especially Thailand and Singapore. This proposition is further strengthened with the presence of the YLF gene cluster and *B*. *pseudomallei* BimA gene allele, two common features of Southeast Asian strains. Phylogenetic clustering and genetic relatedness with Malaysian animal/environmental isolates also showed an association that implicates agriculture and domestication activities as a predisposing factor for infection. The ST1342 is a singleton and was previously reported in Malaysia during an environment linked outbreak in the state of Pahang [3]. This ST has not been reported elsewhere in the world and may be unique to Malaysia. Interestingly, it is also the only sequence type which is LPS type B and is closely linked with the Oceania endemic countries.

Previous studies have reported no relationship between clinical outcome and genotype in human melioidosis cases [17,28]. However in this study, we observed that ST289 and ST658 are resistant to antimicrobials routinely used during the eradication stage [29,30]. ST658 is only found in Sarawak while ST289 has been identified from 6 states in Peninsular Malaysia. The antibiotic treatment protocol for melioidosis in both Peninsular and Borneo Malaysia (Sabah and Sarawak) adheres to the National Antibiotics Guidelines by the Ministry of Health Malaysia (National Antimicrobial Guideline 2019, 3rd Edition). Although 90.5% of the isolates are sensitive to the antimicrobials tested, the observed resistance pattern for ST289 and ST658 isolates may be attributed to any of the intrinsic resistance mechanisms reported for *B*. *pseudomallei* such as reduced drug permeation due to LPS modifications, active efflux, enzymatic inactivation, target alteration and modifications and drug sequestration. This resistance profile obviously limits treatment options and is further challenged by further potential acquired resistance due to the pathogen’s highly recombinogenic nature [30].

Currently, in all microbiology laboratories in Malaysian government hospitals, the interpretive criteria for susceptibility or resistance of *B*. *pseudomallei* using E-tests are based on the Clinical and Laboratory Standards Institute (CLSI) recommendations. Based on the CLSI-based interpretations of the E-tests data, almost 90% of *B*. *pseudomallei* isolated from Malaysian melioidosis patients are sensitive to antimicrobials [31]. Whilst the E-tests interpretations should preferably be confirmed using the broth microdilution method, similar susceptibility rates were also reported by other studies from Thailand [32] and Bangladesh [33].

The MLST approach, although widely applied to determine the clonal relationship among strains, is based on only seven housekeeping genes, and therefore, does not offer high resolution. *B*. *pseudomallei* has high genome plasticity due to frequent recombination that increases genetic divergence contributing to strain-to-strain variation [34] which limits the interpretation of the phylogeographic analysis. This limitation could be circumvented with the analysis of whole genome sequences (WGS) of these local isolates. WGS data will enable variant calling which includes single nucleotide polymorphisms (SNPs), insertions and deletions (indels) to increase bootstrap support and prevent phylogenetic incongruence. Furthermore, identification of structural variants would provide higher resolution with more robust and accurate identification of the origin of the strains. Taken together, WGS data should unravel the genetic mechanisms conferring resistance of ST289 and ST658 to guide the design of effective treatment protocols for melioidosis patients infected with resistant strains. A further limitation of this study is the lack of environmental isolates from locations corresponding to the clinical cases to accurately determine the distribution of STs in Malaysia. A more extensive surveillance program for melioidosis in Malaysia could identify the number of individuals at risk, environmental risk factors, socio economic impact and epidemiological distribution of the bacteria.

## Conclusion

The present study revealed that B. pseudomallei isolated in Malaysia is less heterogenous when compared to isolates from other countries and closely resembled strains from Thailand. Although most of the strains belong to certain common STs already reported in other countries, we identified strains that are unique within Malaysia especially with the discovery of a novel ST. We also noted a potential correlation between sequence types and antimicrobial resistance. Nevertheless, WGS and SNP typing would be able to provide more insightful information on the relationship of the Malaysian B. pseudomallei isolates to their origin, antimicrobial susceptibility and virulence to provide more complete records of B. pseudomallei infection hotspots and genetic diversity. This information will guide the Ministry of Health Malaysia and infectious disease physicians in formulating guidelines for diagnosis, treatment and surveillance of melioidosis in Malaysia.

## Supporting information

S1 Table Demographics data on the 84 *B*. *pseudomallei* isolates from Malaysia. (DOCX)

S2 Table Distribution of sequence types, antimicrobial susceptibility profiles and epidemiological markers among septicaemic melioidosis isolates from Malaysia. (DOCX)
