# Supplementary material for: Multi locus sequence typing of clinical Burkholderia pseudomallei isolates from Malaysia
Source: PLoS Negl Trop Dis. 2020 Dec 28;14(12):e0008979. doi: 10.1371/journal.pntd.0008979 (PMC7793247; doi:10.1371/journal.pntd.0008979)
Supplement: S1 Table — (DOCX) [file pntd.0008979.s001.docx]

**Supplementary Data 1**

**Demographics Data on the 84 *B. pseudomallei* isolates from Malaysia**

| Isolate | Year | Diagnosis | Location | Gender | Age | Ethnicity | Occupation |
| --- | --- | --- | --- | --- | --- | --- | --- |
| BP1 | 2018 | Melioidosis | Perak | Male | 56 | Malay | Farmer |
| BP3 | 2018 | Melioidosis | Negeri Sembilan | Male | 41 | Malay | Unknown |
| BP13 | 2018 | Melioidosis | Sarawak | Male | 7 | Malay | Unknown |
| BP5 | 2018 | Septicaemia | Negeri Sembilan | Male | 35 | Malay | Farmer |
| BP2 | 2018 | Septicaemia | Negeri Sembilan | Male | 76 | Malay | Unknown |
| BP4 | 2018 | Melioidosis | Negeri Sembilan | Male | 37 | Indian | Lorry driver |
| BP10 | 2018 | Pneumonia | Kedah | Male | 86 | Indian | Retired |
| BP11 | 2018 | Melioidosis | Perlis | Male | 47 | Malay | Gardener |
| BP12 | 2018 | Melioidosis | Kelantan | Female | 39 | Malay | Housewife |
| BP14 | 2018 | Septicaemia | Johor | Male | Unknown | Unknown | Farmworker |
| BP15 | 2018 | Melioidosis | Kedah | Male | 50 | Malay | Gardener |
| BP16 | 2018 | Septicaemia | Kelantan | Male | Unknown | Malay | Farmworker |
| BP17 | 2018 | Melioidosis | Kedah | Female | Unknown | Malay | Unknown |
| BP19 | 2018 | Septicaemia | Sarawak | Male | 24 | Unknown | Labourer |
| BP22 | 2018 | Melioidosis | Sabah | Male | 16 | Malay | Unknown |
| BP23 | 2018 | Melioidosis | Sabah | Female | Unknown | Dusun | Unknown |
| BP25 | 2018 | Melioidosis | Kedah | Male | 66 | Malay | Farmer |
| BP26 | 2018 | Septicaemia | Kedah | Male | Unknown | Malay | Farmer |
| BP29 | 2018 | Septicaemia | Kelantan | Male | 40 | Malay | Gardener |
| BP30 | 2018 | Melioidosis | Sabah | Male | Unknown | Iban | Unknown |
| BP31 | 2018 | Pneumonia | Sabah | Female | 27 | Chinese | Mechanic |
| BP32 | 2018 | Melioidosis | Sabah | Male | 31 | Bidayuh | Farmworker |
| BP33 | 2018 | Melioidosis | Sabah | Female | 92 | Iban | Not working |
| BP35 | 2018 | Septicaemia | Sabah | Male | 40 | Chinese | Teacher |
| BP37 | 2018 | Septicaemia | Kelantan | Male | 41 | Malay | Clerk |
| BP39 | 2018 | Melioidosis | Perak | Male | 52 | Malay | Lorry driver |
| BP40 | 2018 | Pneumonia | Pulau Pinang (Penang) | Male | 48 | Malay | Gardener |
| BP41 | 2018 | Melioidosis | Pulau Pinang | Male | 66 | Malay | Retired |
| BP42 | 2018 | Pneumonia | Johor | Female | 30 | Chinese | Unknown |
| BP48 | 2018 | Melioidosis | Johor | Male | Unknown | Malay | Army Personnel |
| BP51 | 2018 | Pneumonia | Negeri Sembilan | Male | 31 | Malay | Farmworker |
| BP53 | 2018 | Melioidosis | Negeri Sembilan | Male | 61 | Malay | Retired |
| BP63 | 2018 | Pneumonia | Terengganu | Male | 80 | Malay | Not working |
| BP64 | 2018 | Melioidosis | Pulau Pinang | Male | 60 | Chinese | Retired |
| BP65 | 2018 | Septicaemia | Johor | Female | Unknown | Unknown | Unknown |
| BP66 | 2018 | Melioidosis | Pahang | Male | Unknown | Malay | Labourer |
| BP67 | 2018 | Melioidosis | Pahang | Male | Unknown | Malay | Technician |
| BP68 | 2018 | Septicaemia | Perak | Male | 52 | Malay | Gardener |
| BP69 | 2018 | Septicaemia | Pulau Pinang | Male | 42 | Indian | Driver |
| BP76 | 2018 | Melioidosis | Selangor | Male | 68 | Chinese | Retired |
| BP77 | 2018 | Pneumonia | Terengganu | Female | 57 | Malay | Fisherman |
| BP84 | 2018 | Melioidosis | Johor | Male | Unknown | Malay | Fishmonger |
| BP85 | 2018 | Melioidosis | Terengganu | Male | 44 | Malay | Farmworker |
| BP86 | 2018 | Septicaemia | Terengganu | Female | Unknown | Malay | Unknown |
| BP87 | 2018 | Pneumonia | Kuala Lumpur | Male | 53 | Chinese | Sales person |
| BP89 | 2018 | Melioidosis | Johor | Male | 69 | Malay | Retired |
| BP92 | 2018 | Septicaemia | Kedah | Male | Unknown | Indian | Unknown |
| BP107 | 2019 | Melioidosis | Melaka | Female | Unknown | Malay | Unknown |
| BP108 | 2019 | Melioidosis | Melaka | Male | 46 | Malay | Policeman |
| BP109 | 2019 | Septicaemia | Melaka | Female | 58 | Indian | Security guard |
| BP110 | 2019 | Septicaemia | Melaka | Male | Unknown | Unknown | Unknown |
| BP111 | 2019 | Melioidosis | Melaka | Male | 42 | Chinese | Legal assistant |
| BP113 | 2019 | Pneumonia | Melaka | Male | 54 | Malay | Estate worker |
| BP116 | 2019 | Melioidosis | Sarawak | Male | 72 | Bidayuh | Not working |
| BP117 | 2019 | Melioidosis | Sarawak | Female | 59 | Unknown | Unknown |
| BP121 | 2019 | Septicaemia | Terengganu | Male | Unknown | Malay | Labourer |
| BP123 | 2019 | Pneumonia | Selangor | Female | 60 | Indian | Retired |
| BP152 | 2019 | Melioidosis | Kelantan | Male | 72 | Malay | Gardener |
| BP158 | 2019 | Septicaemia | Johor | Male | Unknown | Indian | Unknown |
| BP160 | 2019 | Melioidosis | Pahang | Male | Unknown | Malay | Farmer |
| BP161 | 2019 | Melioidosis | Pahang | Female | 59 | Malay | Housewife/Farmer |
| BP164 | 2019 | Septicaemia | Selangor | Male | Unknown | Chinese | Unknown |
| BP165 | 2019 | Septicaemia | Selangor | Male | 49 | Indian | Odd job |
| BP168 | 2019 | Melioidosis | Perak | Male | 47 | Indian | Palm oil estate worker |
| BP169 | 2019 | Pneumonia | Pulau Pinang | Male | Unknown | Malay | Unknown |
| BP173 | 2019 | Melioidosis | Sarawak | Male | 61 | Iban | Not working |
| BP176 | 2019 | Melioidosis | Pahang | Male | 50 | Malay | Agriculture land owner |
| BP178 | 2019 | Septicaemia | Selangor | Female | Unknown | Malay | Housewife |
| BP183 | 2019 | Septicaemia | Perlis | Female | Unknown | Malay | Unknown |
| BP188 | 2019 | Pneumonia | Perak | Male | 44 | Indian | Palm oil estate worker |
| BP190 | 2019 | Melioidosis | Pahang | Male | 45 | Malay | Gardener |
| BP192 | 2019 | Septicaemia | Pahang | Female | Unknown | Orang asli | Housewife |
| BP193 | 2019 | Melioidosis | Sarawak | Male | 60 | Iban | Timber logger |
| BP194 | 2019 | Melioidosis | Perlis | Male | 71 | Unknown | Tour guide |
| BP195 | 2019 | Septicaemia | Pahang | Female | Unknown | Malay | Plantation |
| BP197 | 2019 | Septicaemia | Perak | Male | 62 | Indian | Lorry driver |
| BP202 | 2019 | Melioidosis | Perak | Female | Unknown | Malay | Unknown |
| BP204 | 2019 | Pneumonia | Selangor | Male | 50 | Indian | Army personnel |
| BP210 | 2019 | Melioidosis | Kuala Lumpur | Male | 63 | Malay | Lorry driver |
| BP211 | 2019 | Septicaemia | Johor | Male | 52 | Chinese | Wet market worker |
| BP301 | 2019 | Septicaemia | Selangor | Male | 63 | Indian | Driver |
| BP302 | 2019 | Melioidosis | Kelantan | Male | 52 | Malay | Lorry driver |
| BP303 | 2019 | Pneumonia | Kelantan | Male | 56 | Malay | Gardener |
| BP304 | 2019 | Melioidosis | Perlis | Male | 53 | Malay | Chicken farm worker |
