# Supplementary material for: Multi locus sequence typing of clinical Burkholderia pseudomallei isolates from Malaysia
Source: PLoS Negl Trop Dis. 2020 Dec 28;14(12):e0008979. doi: 10.1371/journal.pntd.0008979 (PMC7793247; doi:10.1371/journal.pntd.0008979)
Supplement: S2 Table — (DOCX) [file pntd.0008979.s002.docx]

**Supplementary Table 2**

**Distribution of sequence types, antimicrobial susceptibility profiles and epidemiological markers among septicaemic melioidosis isolates from Malaysia**

| Isolate | ST^1^ | AmC | | CAZ | | IPM | | Doxy | | Tet | | SXT | | Epidemiological markers | | | |
| --- | --- | --- | --- | --- | --- | --- | --- | --- | --- | --- | --- | --- | --- | --- | --- | --- | --- |
|  |  |  |  |  |  |  |  |  |  |  |  |  |  | **LPS** | ***YLF*** | **BimA_Bp_** | **BimA_Bm_** |
|  |  | **MIC^2^**  **µg/ml** | | **MIC**  **µg/ml** | | **MIC**  **µg/ml** | | **MIC**  **µg/ml** | | **MIC**  **µg/ml** | | **MIC**  **µg/ml** | |  |  |  |  |
| BP1 | 84 | 3.00 | S | 2.00 | S | 0.5 | S | 1.00 | S | 2.00 | S | 3.00 | S | A | + | + | - |
| BP3 | 46 | 2.00 | S | 0.75 | S | 0.25 | S | 0.75 | S | 3.00 | S | 0.38 | S | A | + | + | - |
| BP13 | 658 | 1.50 | S | 1.00 | S | 0.25 | S | 1.00 | S | 1.50 | S | 0.094 | S | A | + | + | - |
| BP5 | 1747 | 1.50 | S | 0.75 | S | 0.25 | S | 0.75 | S | 2.00 | S | 0.125 | S | A | + | + | - |
| BP2 | 51 | 1.50 | S | 0.75 | S | 0.25 | S | 0.75 | S | 2.00 | S | 0.094 | S | A | + | + | - |
| BP4 | 438 | 2.00 | S | 1.00 | S | 0.38 | S | 0.75 | S | 3.00 | S | 2.00 | S | A | + | + | - |
| BP10 | 289 | 2.00 | S | 2.00 | S | 0.38 | S | 2.00 | S | 3.00 | S | 0.064 | S | A | + | + | - |
| BP11 | 1742 | 1.50 | S | 0.75 | S | 0.25 | S | 0.75 | S | 2.00 | S | 0.125 | S | A | + | + | - |
| BP12 | 1546 | 1.50 | S | 1.00 | S | 0.25 | S | 1.00 | S | 1.50 | S | 0.094 | S | A | + | + | - |
| BP14 | 289 | 3.00 | S | 1.00 | S | 0.50 | S | 1.50 | S | 32.00 | R | 0.094 | S | A | + | + | - |
| BP15 | 84 | 3.00 | S | 1.50 | S | 0.75 | S | 0.75 | S | 1.50 | S | 2.00 | S | A | + | + | - |
| BP16 | 1057 | 2.00 | S | 1.00 | S | 0.38 | S | 0.75 | S | 3.00 | S | 2.00 | S | A | + | + | - |
| BP17 | 84 | 3.00 | S | 1.00 | S | 0.75 | S | 1.00 | S | 4.00 | S | 2.00 | S | A | + | + | - |
| BP19 | 658 | 1.50 | S | 2.00 | S | 0.38 | S | 1.50 | S | 2.00 | S | 6.00 | R | A | + | + | - |
| BP22 | 84 | 4.00 | S | 0.75 | S | 0.19 | S | 1.50 | S | 2.00 | S | 0.32 | S | A | + | + | - |
| BP23 | 50 | 4.00 | S | 1.00 | S | 0.38 | S | 0.75 | S | 1.50 | S | 0.50 | S | A | + | + | - |
| BP25 | 1702 | 3.00 | S | 3.00 | S | 0.75 | S | 1.00 | S | 3.00 | S | 1.50 | S | A | + | + | - |
| BP26 | 288 | 3.00 | S | 2.00 | S | 0.38 | S | 0.38 | S | 2.00 | S | 6.00 | R | A | + | + | - |
| BP29 | 54 | 2.00 | S | 1.00 | S | 0.25 | S | 1.00 | S | 1.50 | S | 0.125 | S | A | + | + | - |
| BP30 | 50 | 4.00 | S | 2.00 | S | 0.25 | S | 0.75 | S | 0.75 | S | 0.19 | S | A | + | + | - |
| BP31 | 50 | 4.00 | S | 1.00 | S | 0.38 | S | 0.75 | S | 1.00 | S | 0.50 | S | A | + | + | - |
| BP32 | 84 | 4.00 | S | 1.00 | S | 0.25 | S. | 0.75 | S | 1.50 | S | 0.047 | S | A | + | + | - |
| BP33 | 58 | 4.00 | S | 1.00 | S | 0.25 | S | 0.75 | S | 1.50 | S | 0.125 | S | A | + | + | - |
| BP35 | 84 | 4.00 | S | 0.75 | S | 0.38 | S | 0.75 | S | 2.00 | S | 0.19 | S | A | + | + | - |
| BP37 | 507 | 1.50 | S | 1.50 | S | 0.38 | S | 0.50 | S | 1.50 | S | 0.16 | S | A | + | + | - |
| BP39 | 51 | 48.00 | R | 1.50 | S | 0.38 | S | 0.50 | S | 24.00 | R | 16.00 | R | A | + | + | - |
| BP40 | 46 | 2.00 | S | 3.00 | S | 0.25 | S | 0.75 | S | 1.50 | S | 0.38 | S | A | + | + | - |
| BP41 | 376 | 2.00 | S | 1.50 | S | 0.38 | S | 0.50 | S | 1.00 | S | 0.25 | S | A | + | + | - |
| BP42 | 46 | 2.00 | S | 1.00 | S | 0.50 | S | 1.00 | S | 4.00 | S | 0.19 | S | A | + | + | - |
| BP48 | 84 | 1.50 | S | 0.75 | S | 0.38 | S | 0.75 | S | 1.50 | S | 0.094 | S | A | + | + | - |
| BP51 | 54 | 2.00 | S | 1.50 | S | 0.50 | S | 1.50 | S | 3.00 | S | 2.00 | S | A | + | + | - |
| BP53 | 1342 | 1.50 | S | 3.00 | S | 0.25 | S | 0.75 | S | 1.50 | S | 0.19 | S | B | + | + | - |
| BP63 | 289 | 4.00 | S | 1.00 | S | 0.50 | S | 0.75 | S | 4.00 | S | 12.00 | R | A | + | + | - |
| BP64 | 289 | 32.00 | R | 1.50 | S | 0.25 | S | 0.75 | S | 16.00 | R | 4.00 | R | A | + | + | - |
| BP65 | 84 | 2.00 | S | 1.00 | S | 0.25 | S | 0.75 | S | 1.00 | S | 0.047 | S | A | + | + | - |
| BP66 | 97 | 1.50 | S | 1.00 | S | 0.25 | S | 1.00 | S | 3.00 | S | 0.032 | S | A | + | + | - |
| BP67 | 1342 | 1.00 | S | 0.75 | S | 0.38 | S | 1.50 | S | 1.00 | S | 0.016 | S | B | + | + | - |
| BP68 | 84 | 2.00 | S | 1.00 | S | 0.25 | S | 0.75 | S | 1.00 | S | 3.00 | S | A | + | + | - |
| BP69 | 46 | 2.00 | S | 3.00 | S | 0.50 | S | 0.75 | S | 1.50 | S | 5.00 | R | A | + | + | - |
| BP76 | 1057 | 4.00 | S | 1.00 | S | 0.19 | S | 1.00 | S | 1.00 | S | 0.38 | S | A | + | + | - |
| BP77 | 54 | 4.00 | S | 1.00 | S | 0.50 | S | 1.50 | S | 2.00 | S | 0.125 | S | A | + | + | - |
| BP84 | 51 | 1.50 | S | 1.00 | S | 0.50 | S | 1.00 | S | 1.00 | S | 0.032 | S | A | + | + | - |
| BP85 | 366 | 4.00 | S | 2.00 | S | 0.50 | S | 1.00 | S | 4.00 | S | 0.75 | S | A | + | + | - |
| BP86 | 54 | 4.00 | S | 1.00 | S | 0.50 | S | 1.50 | S | 4.00 | S | 0.125 | S | A | + | + | - |
| BP87 | 51 | 4.00 | S | 4.00 | S | 0.75 | S | 0.75 | S | 2.00 | S | 0.19 | S | A | + | + | - |
| BP89 | 423 | 1.50 | S | 1.00 | S | 0.50 | S | 1.00 | S | 1.00 | S | 0.125 | S | A | + | + | - |
| BP92 | 54 | 2.00 | S | 3.00 | S | 0.38 | S | 1.00 | S | 2.00 | S | 0.5 | S | A | + | + | - |
| BP107 | 46 | 2.00 | S | 1.50 | S | 0.38 | S | 1.00 | S | 1.00 | S | 0.125 | S | A | + | + | - |
| BP108 | 1743 | 2.00 | S | 0.75 | S | 0.38 | S | 0.75 | S | 0.75 | S | 2.00 | S | A | + | + | - |
| BP109 | 289 | 2.00 | S | 0.75 | S | 0.38 | S | 0.38 | S | 0.50 | S | 1.50 | S | A | + | + | - |
| BP110 | 46 | 2.00 | S | 1.5 | S | 0.38 | S | 1.00 | S | 1.00 | S | 1.00 | S | A | + | + | - |
| BP111 | 56 | 2.00 | S | 1.00 | S | 0.38 | S | 0.75 | S | 1.00 | S | 2.00 | S | A | + | + | - |
| BP113 | 51 | 2.00 | S | 1.00 | S | 0.25 | S | 0.5 | S | 1.50 | S | 1.5 | S | A | + | + | - |
| BP116 | 1744 | 4.00 | S | 1.00 | S | 0.75 | S | 1.00 | S | 2.00 | S | 0.09 | S | A | + | + | - |
| BP117 | 881 | 6.00 | S | 0.75 | S | 0.38 | S | 1.50 | S | 1.50 | S | 0.125 | S | A | + | + | - |
| BP121 | 54 | 4.00 | S | 2.00 | S | 0.50 | S | 2.00 | S | 4.00 | S | 0.09 | S | A | + | + | - |
| BP123 | 84 | 3.00 | S | 1.00 | S | 0.50 | S | 1.00 | S | 1.50 | S | 0.023 | S | A | + | + | - |
| BP152 | 54 | 1.50 | S | 1.50 | S | 0.38 | S | 0.75 | S | 1.00 | S | 0.094 | S | A | + | + | - |
| BP158 | 1745 | 1.50 | S | 1.00 | S | 0.50 | S | 1.00 | S | 1.00 | S | 0.125 | S | A | + | + | - |
| BP160 | 54 | 1.50 | S | 0.75 | S | 0.50 | S | 0.75 | S | 2.00 | S | 0.25 | S | A | + | + | - |
| BP161 | 1342 | 2.00 | S | 0.75 | S | 0.38 | S | 0.75 | S | 1.50 | S | 0.47 | S | B | + | + | - |
| BP164 | 51 | 4.00 | S | 1.00 | S | 0.25 | S | 1.50 | S | 1.50 | S | 1.50 | S | A | + | + | - |
| BP165 | 376 | 1.50 | S | 3.00 | S | 0.25 | S | 0.75 | S | 3.00 | S | 6.00 | R | A | + | + | - |
| BP168 | 54 | 2.00 | S | 1.50 | S | 0.38 | S | 1.00 | S | 1.50 | S | 0.70 | S | A | + | + | - |
| BP169 | 46 | 3.00 | S | 3.00 | S | 0.38 | S | 0.50 | S | 1.50 | S | 0.19 | S | A | + | + | - |
| BP173 | 1746 | 4.00 | S | 1.00 | S | 0.25 | S | 2.00 | S | 2.00 | S | 0.70 | S | A | + | + | - |
| BP176 | 1342 | 1.50 | S | 1.00 | S | 0.25 | S | 1.00 | S | 1.50 | S | 0.094 | S | B | + | + | - |
| BP178 | 51 | 2.00 | S | 1.00 | S | 0.25 | S | 2.00 | S | 0.75 | S | 0.125 | S | A | + | + | - |
| BP183 | 46 | 3.00 | S | 3.00 | S | 0.5 | S | 1.50 | S | 3.00 | S | 0.125 | S | A | + | + | - |
| BP188 | 54 | 1.50 | S | 3.00 | S | 0.5 | S | 1.50 | S | 4.00 | S | 2.00 | S | A | + | + | - |
| BP190 | 54 | 1.50 | S | 1.00 | S | 0.38 | S | 1.50 | S | 3.00 | S | 1.90 | S | A | + | + | - |
| BP192 | 438 | 1.5 | S | 0.75 | S | 0.25 | S | 1.00 | S | 1.50 | S | 0.016 | S | A | + | + | - |
| BP193 | 658 | 4.00 | S | 2.25 | S | 3.00 | S | 2.00 | S | 2.00 | S | 8.00 | R | A | + | + | - |
| BP194 | 46 | 4.00 | S | 1.50 | S | 0.5 | S | 1.00 | S | 1.50 | S | 0.023 | S | A | + | + | - |
| BP195 | 84 | 1.50 | S | 1.00 | S | 0.25 | S | 1.50 | S | 4.00 | S | 0.127 | S | A | + | + | - |
| BP197 | 1057 | 1.50 | S | 3.00 | S | 1 | S | 2.00 | S | 4.00 | S | 2.00 | S | A | + | + | - |
| BP202 | 1057 | 2.00 | S | 2.00 | S | 0.38 | S | 1.00 | S | 2.00 | S | 0.19 | S | A | + | + | - |
| BP204 | 54 | 3.00 | S | 1.00 | S | 0.5 | S | 1.50 | S | 1.50 | S | 0.023 | S | A | + | + | - |
| BP210 | 46 | 48.00 | R | 1.00 | S | 0.5 | S | 1.50 | S | 16.00 | R | 6.00 | R | A | + | + | - |
| BP211 | 84 | 3.00 | S | 1.00 | S | 0.25 | S | 0.75 | S | 1.50 | S | 1.50 | S | A | + | + | - |
| BP301 | 51 | 1.50 | S | 3.00 | S | 1 | S | 2.00 | S | 4.00 | S | 2.00 | S | A | + | + | - |
| BP302 | 371 | 4.00 | S | 2.00 | S | 0.5 | S | 1.00 | S | 4.00 | S | 0.38 | S | A | + | + | - |
| BP303 | 289 | 2.00 | S | 1.00 | S | 0.38 | S | 1.50 | S | 4.00 | S | 1.50 | S | A | + | + | - |
| BP304 | 1468 | 4.00 | S | 1.00 | S | 0.5 | S | 1.50 | S | 2.00 | S | 0.125 | S | A | + | + | - |

^1^Abbreviations used in the table:

| ST | Sequence Type |  | IPM | Imipenem |  | LPS | Lipopolysaccharide |
| --- | --- | --- | --- | --- | --- | --- | --- |
| S | Sensitive |  | Doxy | Doxycycline |  | *YLF* | *Yersinia* like fimbriae gene |
| R | Resistant |  | Tet | Tetracycline |  | BimA_Bp_ | *Burkholderia pseudomallei* BimA allele |
| AmC | Amoxicillin clauvulanate |  | SXT | Trimethoprim-sulfamethaxazole |  | BimA_Bm_ | *Burkholderia mallei* BimA allele |
| CAZ | Ceftazidime |  |  |  |  |  |  |

^2^MIC determination and interpretation are based on the Clinical & Laboratory Standards Institute (CLSI, 2017) Guidelines (M45, 3^rd^ edition; Table 21 (page 60-63)
